# Polarization dependence of second harmonic generation from plasmonic nanoprism arrays

**DOI:** 10.1038/s41598-019-47970-3

**Published:** 2019-08-08

**Authors:** K. Y. Raygoza-Sánchez, I. Rocha-Mendoza, P. Segovia, A. V. Krasavin, G. Marino, T. Cesca, N. Michieli, G. Mattei, A. V. Zayats, R. Rangel-Rojo

**Affiliations:** 1 Maestría y Posgrado en Ciencias, Universidad Autónoma de Baja California, Carretera Transpeninsular 3917, 22860 Ensenada, B.C. Mexico; 2 Optics Department, Centro de Investigación Científica y de Educación Superior de Ensenada, Carretera Ensenada-Tijuana, No. 3918, Zona Playitas, 22860 Ensenada, B.C. Mexico; 3 Researcher of Cátedras CONACYT Centro de Investigación Científica y de Educación Superior de Ensenada, Carretera Ensenada-Tijuana, No. 3918, Zona Playitas, 22860 Ensenada, B.C. Mexico; 4 Department of Physics and London Centre for Nanotechnology King’s College London, Strand, London, WC2R 2LS UK; 5 Dipartimento di Fisica e Astronomia Galileo Galilei, Università degli Studi di Padova, Via Marzolo 8, 35131 Padova, Italy

**Keywords:** Metamaterials, Nanoscale materials

## Abstract

The second order nonlinear optical response of gold nanoprisms arrays is investigated by means of second harmonic generation (SHG) experiments and simulations. The polarization dependence of the nonlinear response exhibits a 6-fold symmetry, attributed to the local field enhancement through the excitation of the surface plasmon resonances in bow-tie nanoantennas forming the arrays. Experiments show that for polarization of the input light producing excitation of the plasmonic resonances in the bow-tie nanoantennas, the SHG signal is enhanced; this despite the fact that the linear absorption spectrum is not dependent on polarization. The results are confirmed by electrodynamic simulations which demonstrate that SHG is also determined by the local field distribution in the nanoarrays. Moreover, the maximum of SHG intensity is observed at slightly off-resonance excitation, as implemented in the experiments, showing a close relation between the polarization dependence and the structure of the material, additionally revealing the importance of the presence of non-normal electric field components as under focused beam and oblique illumination.

## Introduction

Nanocomposite materials, containing dielectric, semiconductor or metallic nanoparticles with typical sizes in the 1–100 nm range, have attracted considerable interest for their optical properties, and potential applications, such as optical signal processing^1^, and chemical or biological sensing^2–4^. When the distribution of the nanoparticles is random or they are structured in subwavelength arrays and diffraction effects are absent, the nanocomposite is perceived by light as a uniform effective medium, for which a term of ‘metamaterial’ or ‘metasurface’ was coined. The main feature of such metamaterials is the capability of tailoring their optical properties by the manipulation of their structure: nanoparticle ‘meta-atom’ composition, dielectric contrast, shape, orientation and spatial distribution are some of the parameters that can be varied to engineer and optimize their optical response.

Metamaterials composed of metallic nanostructures have the  additional advantage that due to their plasmonic nature, the incident electromagnetic field can be concentrated into very small regions around or between the meta-atoms, and thus greatly enhanced field amplitudes can be produced. This local field enhancement can be exploited to significantly increase the materials nonlinear optical properties, or for chemical and biological sensing through enhanced fluorescence or Raman signals^5–7^. The third-order nonlinear response of these materials, manifested through effects such as nonlinear absorption and refraction, has been extensively studied, mainly for metallic disordered systems either embedded in glass or in solution^8,9^, but also in more ordered structures^10,11^.

Although macroscopically centrosymmetric, these nanocomposites present interfaces that break the symmetry, and therefore allow the observation of second-order nonlinear optical effects such as SHG. These are usually surface effects, where the contribution comes only from a very thin layer of material at either side of the interface. For nanoparticles, however, given their small dimensions, this can include most of its bulk, and again, local field enhancement can boost the SHG signal produced in these materials to significant levels^12^. While effective second-order susceptibilities are comparable to those of nonlinear crystals^13^, it is important to notice that since the interaction volumes are usually very small, the overall SHG response cannot be expected to be comparable to that of phase-matched bulk noncentrosymmetric media, with interaction lengths in a millimetre to centimetre range. However, the metallic nonlinearity and efficiency of SHG is very high^12^ and furthermore, since SHG is very sensitive to the symmetry of the sample structure, SHG can be seen as a tool for studying the nanoscopic morphology of the materials^14,15^. In particular, this approach has been applied to a system consisting of elongated metallic nanoparticles embedded in glass and aligned along a preferential direction, for which a very close relation of the measured SHG signal with the light polarization, sample orientation, and structure was established^14,16^.

Recently, third-order nonlinear optical properties of metasurfaces consisting of ordered arrays of nanoprisms have been thoroughly studied^17–19^. Placing the nanoelements, in this case triangularly shaped nanoprisms, in an ordered array with a well-defined geometry allows having a response that is the coherent addition of the individual responses of each nanoparticle, which is larger than that of an equivalent disordered nanoparticle assembly. On the other hand, in these materials the field is further enhanced by a nanoantenna effect in a local region between two nanoprisms in a bow-tie configuration, as shown in finite element method (FEM) simulations^19^. The polarization dependence of the nonlinear absorption was correlated to this enhancement and with the symmetry of the sample, with a good agreement between the simulations and the experiment^19^. Therefore it poses an even more interesting question if there is such a correlation for the SHG, which is highly dependent on symmetry, and its relationship to localized surface plasmon resonances (LSPR)^15^, and more recently, to surface lattice resonances^20^. The elucidation of the relation between the field enhancement obtained in these metasurfaces, and their nonlinear response can help on improvements in the design of chemical and biological sensors based on them.

In this article we present an experimental and numerical study of SHG from a honeycomb array of Au nanoprisms and its relation to the polarization of the fundamental light. The observed dependency is correlated to the symmetry properties of the metasurface, which allows accessing a wide variety of the components of the nonlinear susceptibility tensor, and reveals the importance of the focused illumination.

## Experimental

### Metallic nanoprism arrays fabrication

**Figure 1:**
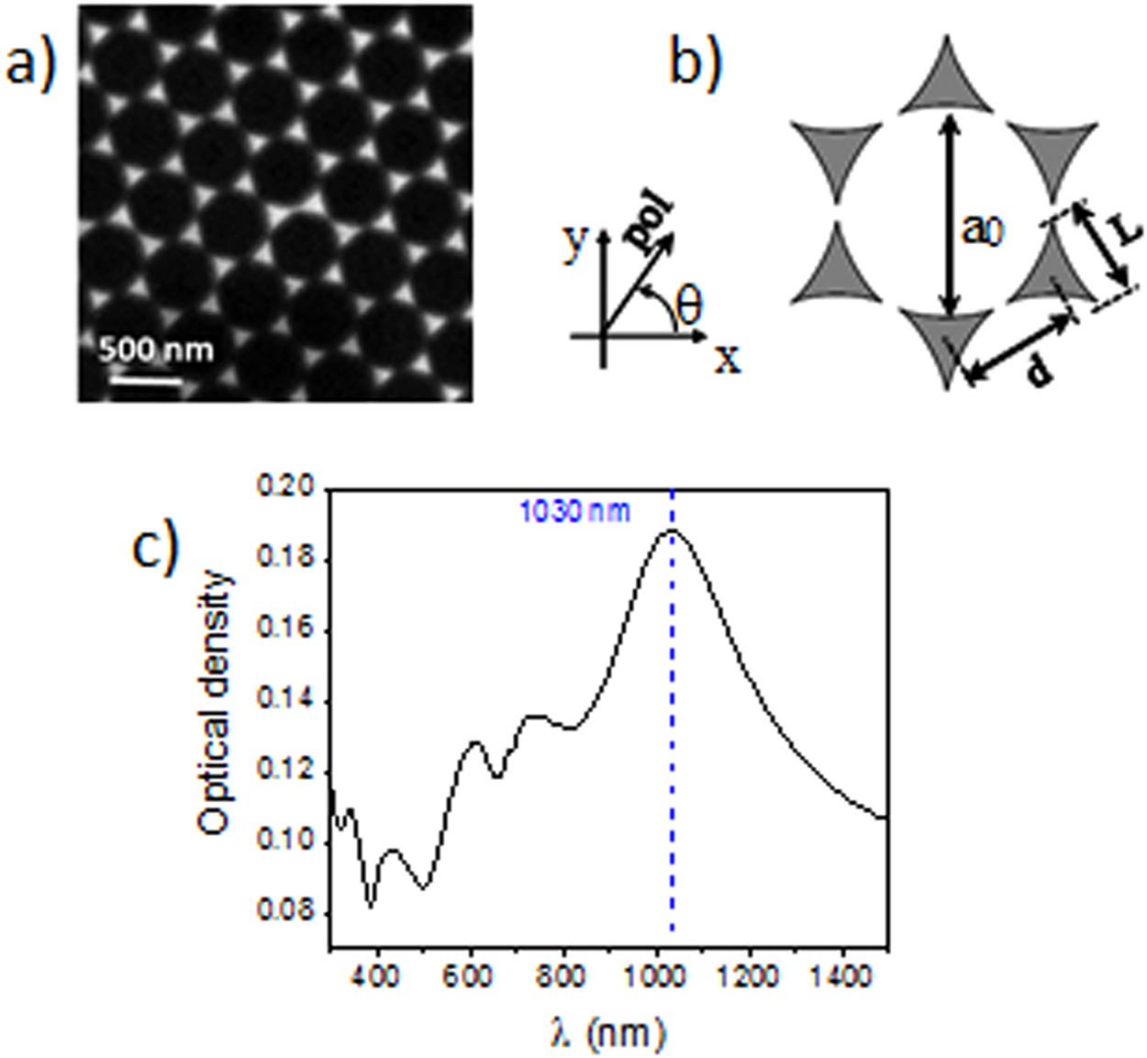
(**a**) SEM image of the nanoprism array, (**b**) array geometry, and (**c**) absorbance spectrum of the structure. The vertical line shows the dipolar LSPR.

Two -dimensional arrays of Au nanoprisms were fabricated using a nanosphere lithography technique. The manufacturing process is described in detail in^21^ and is only briefly outlined here. Polystyrene nanospheres of a 522 nm diameter are first self-assembled on a surface of a clean silica glass substrate, forming a colloidal layer. Afterwards, Au is deposited on the substrate by thermal evaporation. Then, the nanospheres are removed using a solvent. Finally, a silica layer is deposited by magnetron sputtering on the resulting Au nanoprisms to prevent possible oxidation effects and physical damage of the nanoarray. The top protective layer is estimated to be 146 nm thick. The resulting nanostructures, observed using a field emission scanning electron microscope (FE-SEM, model Sigma HD, Zeiss) are shown in Fig. 1a. The geometric parameters of the nanoarray are depicted in Fig. 1b: *α*_0_ = 522 ± 5 nm is the lattice parameter (equal to the polystyrene nanospheres diameter), *d* = 290 ± 9 nm is the distance between the nanoprisms, *L* = 155 ± 3 nm is the side length of each nanoprism and *h* is their height. The latter, measured by atomic force microscopy (AFM, model NT-MDT Nova Solver-PRO in non-contact mode) is found to be *h* = 34 ± 2 nm.

The measured absorption spectrum of the sample taken with unpolarized incident light is shown in Fig. 1c. The spectrum shows a well-defined absorption band centered around 1030 nm, which corresponds to the dipolar localized surface plasmon (LSPR) response of the nanoprisms. The other absorption features at shorter wavelengths correspond to the quadrupolar, and higher order multipolar LSPRs, as it has been shown by FEM simulations previously performed on similar arrays^19^. These studies showed experimentally that the absorption spectrum of the array taken with linearly polarized light does not depend on the polarization angle *θ* measured with respect to the array structure (shown in Fig. 1b), a fact agreeing with symmetry considerations^22^ also corroborated by the simulations^19^.

### SHG experiments

**Figure 2:**
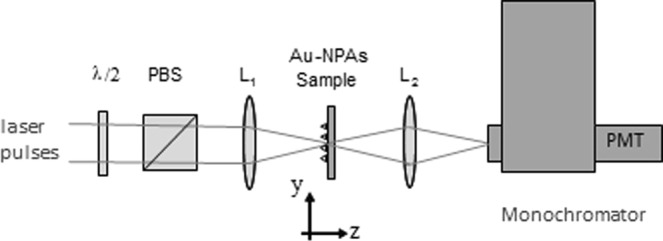
Experimental setup for the measurement of SHG from an array of Au nanoprisms.

Second harmonic generation experiments were conducted in a transmission mode using a focused beam to excite the sample at normal incidence. The schematic experimental setup is shown in Fig. 2. The fundamental light was generated by a Ti:Sapphire oscillator (Coherent Mira 900) pumped at 532 nm. The oscillator produces ultrashort 90 fs linearly polarized pulses at a 76 MHz repetition rate. The pulses had a spectral width of 12 nm and were centered at an 810 nm wavelength. The polarization azimuthal angle θ of the excitation (fundamental) beam was rotated using a *λ/2* wave-plate, in order to study the dependence of the SHG signal on the polarization direction of the fundamental light. The beam, with a 3 mm diameter, was focused onto the sample by means of a *L*_*1*_ = 50 mm focal length lens, down to a 17 μm diameter on the sample surface, resulting in incident peak irradiances of up to 6 GW/cm^2^. Given this spot size, only about 30 unit cells of the array are illuminated during the SHG measurements, so long-range spatial variations of the structure are not important. The SHG signal generated in the forward direction was collected using a second lens of a *L*_*2*_ = 30 mm focal length to image the point spread function at the monochromator entrance. Finally, the signal was detected using a photomultiplier tube (PMT) connected to a current/voltage pre-amplifier circuit and a digital oscilloscope. A polarizer cube (PBS) is located between the *λ/2* wave-plate and the first lens to measure the dependence of SHG on the incident power.

### Numerical modelling

Numerical analysis of the nonlinear interaction of light with the nanoprisms array, in both linear and nonlinear regimes, was performed in a frequency domain using a finite element method (COMSOL Multiphysics software). The nanostructure was illuminated by a plane electromagnetic wave at various angles with respect to the surface normal (the z axis in Fig. 2), also varying the azimuthal polarization angle *θ*. The infinite honeycomb structure of the arrays was modelled by calculating the full vectorial structure of the fields in the corresponding rhomboidal unit cell containing two nanoprisms, using Bloch boundary conditions on its sides. Setting the proper field phase delays on the pairs of the opposite boundaries allowed modelling various incident and azimuthal angles of the incident wave. In the case of the generated second harmonic spectral component the phase delay was set to twice that of the fundamental wave. To avoid field singularities at the sharp edges, which are particularly undesirable in modelling nonlinear optical effects, the corners and the edges of the nanoprisms were rounded with a radius of 10 nm. The top and the bottom boundaries of the simulation domain were interfaced by perfectly matched layers to ensure the absence of back reflections. The refractive index of silica was considered to be constant in the studied frequency range (n = 1.46), while for gold it was taken from^23^.

The SHG generation from the gold nanoprisms array was numerically simulated within an undepleted pump approximation (weak nonlinearity) using a two-step model^24,25^. In the first step the interaction of the fundamental incident wave with the nanostructures was modeled to determine the local electromagnetic fields at the nanoprism surface. In the second step, the local distribution of the obtained fundamental field was used to calculate the nonlinear polarizability of the nanoprisms^24,25^. Gold is a centrosymmetric material, so the second order nonlinear response, requiring symmetry breaking, occurs at the nanostructure boundaries and is related to the anharmonic dynamics of the free electron gas in the field gradients, and screening electron concentration gradients at the nanostructure surface. Generally, such process can be described by the hydrodynamic model^6^, which in the first approximation leads to the treatment of the nonlinear response in the framework of surface nonlinearity^26^, with the leading term of the second harmonic polarization usually considered to be normal to the surface^27^:

1 $${P}_{2{\rm{\omega }},\perp }({\bf{r}})={\chi }_{\perp \perp \perp }{E}_{{\rm{\omega }},\perp }^{2}({\bf{r}}),$$

where *χ*_⊥⊥⊥_ is the corresponding surface nonlinear susceptibility component and *Eω*_,⊥_(*ω*) is the fundamental electric field component, perpendicular to the nanoprism surface. In order to model the surface metallic nonlinearity, a very thin gold surface layer at the boundary of the nanoprisms was considered and the generated nonlinear polarization was implemented in it using Eq. 1. Then, in the second simulation step, the nonlinear polarization obtained was set as a source for the electromagnetic field at the second harmonic frequency, which was calculated in the entire simulation domain, with their power integrated over the bottom (substrate) domain boundary, modelling the generation geometry implemented in the experiments^24,25^.

## Results and Discussion

**Figure 3:**
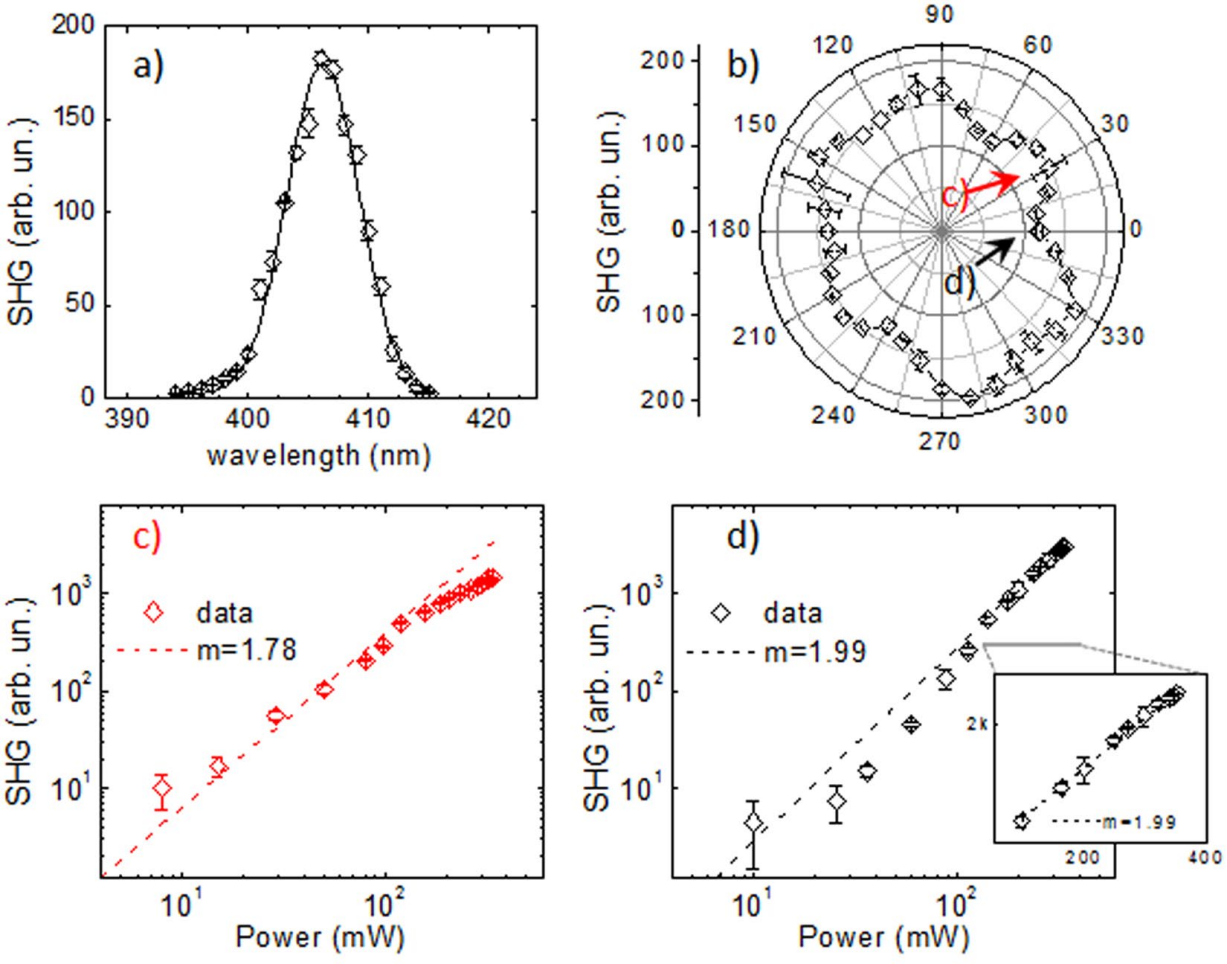
Spectral (**a**) and polarization (**b**) dependences of the SHG signal. Fundamental intensity dependence of SHG signal for polarization angles corresponding to (**c**) a maximum, 30°, and (**d**) a minimum, 0° in this case. The dashed lines in (**c**,**d**) represent quadratic power dependence fits to the data.

The SHG spectrum experimentally measured for the nanoprism array presents a peak centered at a wavelength of 405 nm, and having a width of ~8 nm, as shown in Fig. 3a. This confirms that the measured signal is indeed the second harmonic, as the central wavelength is half that of the input light, and the width is smaller than the original laser width by a factor of $\sqrt{2}$, and no significant broadband two-photon fluorescence was observed.

The polarization dependence of the SHG was measured, with the polarization azimuthal angle defined in relation to the laboratory reference frame as shown in Fig. 1b, i.e. the 0° angle corresponds to the polarization direction along the x-axis. Figure 3b shows a polar chart for the measured polarization dependence of the SHG-signal, taken at an average fundamental power of 88 mW. A clear six-fold symmetry, with SHG maxima every 60° starting from the 30° angle is clearly seen, coinciding with the geometry of the sample. The average ratio between subsequent minima and maxima was found to be approximately ~1.21.

Another test performed was to measure the dependence of the signal on the input irradiance. It was done at two different polarization angles as shown by the red and black arrows in Fig. 3b, corresponding to a SHG maximum and a SHG minimum, respectively. Figure 3c shows the former case, corresponding to the excitation polarized along the bow-tie nanoprism structure. With the data displayed in a log-log scale, it fits very well to a linear relationship with slope 2 for average powers lower than 100 mW (corresponding to *E*_*pulse*_ = 1.3 nJ). This is an indication that we have indeed a second order nonlinear process, as expected for SHG. At average powers higher than 100 mW, a deviation from this behavior can be seen. This could either indicate a ‘saturation’ of the signal, due to a high density of the surface electrons that depletes the harmonic plasmon oscillation, according to Sugita *et al*.^15^, or the onset of damage in the sample^14^, but no conclusive evidence of either could be established. Figure 3d shows the case of the other polarization direction, producing a minimum in the SHG signal. For this angle, the data at low powers seems to have a slope close to 1, indicating the presence of linearly scattered light in the detected signal. At powers higher than 100 mW the signal is well fitted with a line with a slope 2, indicating a clear SHG signal.

**Figure 4:**
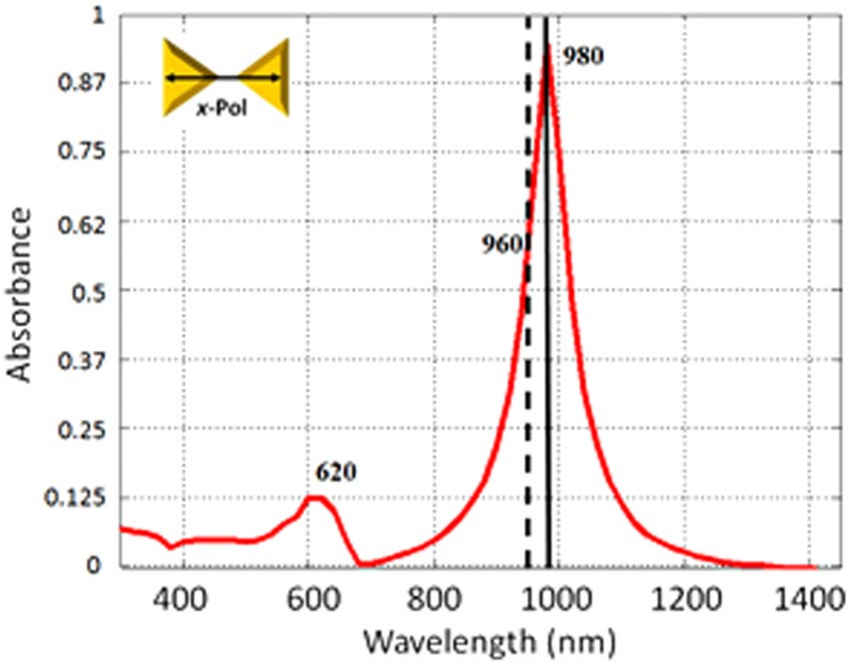
Calculated optical absorbance spectrum of the Au nanoprism array, obtained at normal incidence, showing the plasmonic resonances at 980 and 620 nm.

In the numerical model, the nanoprisms geometrical parameters, determined from SEM and AFM given above, were further optimized to get the best match possible to the experimental absorption spectrum. The simulation was performed considering a plane wave illumination at normal incidence with polarization aligned along the nanoprism symmetry axis, as shown in the inset of Fig. 4. The time-averaged power transmission coefficient *T*_*p*_ is obtained via an *S*-parameter *T*_*p*_ = *|S*_*21*_*|*^*2*^. Using this value it is possible to calculate the absorbance *A* through the relation *T*_*p*_ = *10*^*−A*^, the simulated spectrum is presented in Fig. 4. The fitting parameters used for comparing experimental and theoretical values were *L* = 170 nm and *d* = 300 nm, keeping the height of the nanoprisms fixed at 34 nm. The spectrum shows characteristic absorbance peaks corresponding to the dipolar and quadrupolar resonances of the nanoprisms at 980 and 620 nm wavelengths, respectively.

The agreement in the experimentally measured and calculated peak positions is quite good considering that only a selected set of geometrical parameters was used to reproduce both resonances in the simulations, and they are very close to  those experimentally observed, without changing drastically the original geometry. In addition, it can be observed that calculations exhibit narrow resonance widths in comparison with the experimentally measured. This is probably due to the variability of the nanoprisms geometrical parameters across the actual fabricated array, such as gaps separating prisms and apex shapes.

In order to study the effect of the polarization angle of the incident light on the absorbance spectrum, a set of simulations were carried out for various azimuthal angles. The results show that the shape of the absorption spectrum is independent on the polarization, which is consistent with previously published results^19^. The calculated peak occurs at *λ* = 980 nm, while the experimental peak is at the slightly longer wavelength of 1030 nm, at the same time being considerably broader (Fig. 1c).

In order to take into account this discrepancy in the SHG simulations, and considering that the experiments were not conducted exactly at resonance, but rather above it, the wavelength at which the simulations were conducted was corrected. This is done by keeping in the simulations the same absorbance A(@*λ*_peak_)/A(@*λ*_laser_) ratio measured experimentally, which was A(@1030 nm)/A(@810 nm) = 1.46. The same condition for the simulated absorbance was fulfilled by A(@*λ*_sim_) = A(@980 nm)/1.46, thus the simulations were performed at *λ*_sim_ = 960 nm.

**Figure 5:**
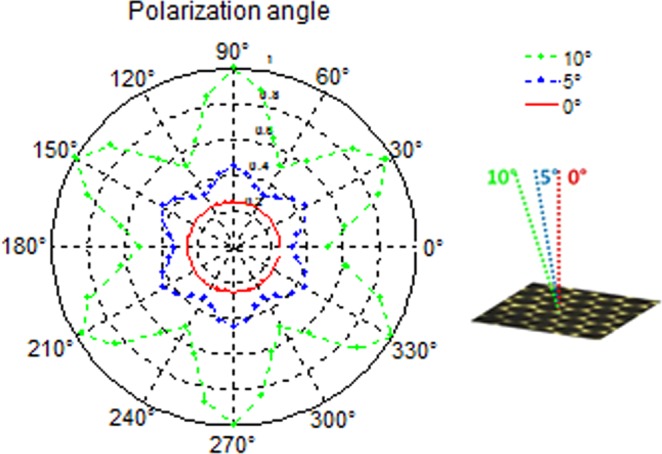
Polar plot of the simulated SHG signal (in a.u.) as a function of the polarization of the excitation field for two different incidence angles: 0° (red points), 5° (blue points) and 10° (green points).

SHG simulations were performed for normal incidence onto the sample for different polarization angles. To analyse the SHG response of the structure as function of the polarization angle of the incident field, the forward radiated SHG output power was calculated using the surface integral of time average power outflow on the surface below the structure. Figure 5 shows the results, with a SHG signal at normal incidence that does not depend on polarization angle *θ* (red line). However, when the SHG signal is calculated for an incident angle different from 0°, a 6-fold polarization angular response is observed, which coincides with the 6-fold symmetry observed in the experimental results (Fig. 3b). Figure 5 shows the results for angles of incidence of 5° and 10°, which indicate that the depth of modulation increases with the incidence angle. One important point to notice here is that the simulations were made assuming plane wave illumination, while the experiments used a weakly focused beam, with a distribution of the incident angles. Thus the facts that no polarization direction dependence was theoretically seen at the normal incidence, and that the experiments coincide well with the simulation at a 5° incidence angle suggest that the observed 6-fold variation is due to the presence of non-normal electric field components in the focused beam, and the complex polarization structure produced in the focal plane. For the beam diameter and focusing conditions employed, the light fills a cone with a 3.4° convergence angle.

**Figure 6:**
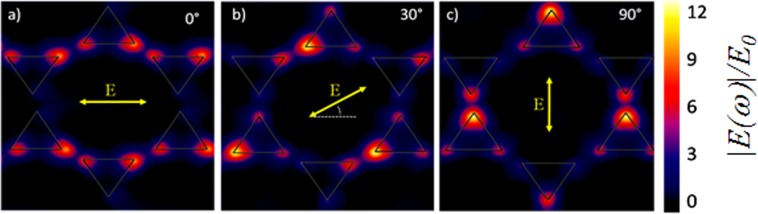
Numerically simulated distribution of the fundamental electric field normalized to the input field, 10 nm above the nanoprisms for polarizations along the symmetry directions of the array: (**a**) 0°, (**b**) 30°, and (**c**) 90° at an incident angle of 5° with respect to the normal to the surface.

The simulated electric field maps corresponding to three different polarization angles of the fundamental light incident obliquely at 5° with respect to the surface normal are presented in Fig. 6. The peak SHG signals at the 30° and 90° polarization angles observed in the experiment (Fig. 3b) are consistent with the electric field enhancement observed in the simulated field maps, in Fig. 6(b,c). In fact, each time the fundamental light polarization is aligned along the symmetry axis of any prism pair in the array, a field enhancement is produced. By contrast, illumination at any other polarization angle will produce a smaller field enhancement, as exemplified in Fig. 6(a) for *θ*  = 0°. The fact that the experimental angular response is somewhat elongated along the 120°–300° direction, can be either due to experimental error, or to the presence of a smaller component of the response with a different symmetry dependence, such as a quadrupolar contribution, but further investigation would be required to clarify this point.

**Figure 7:**
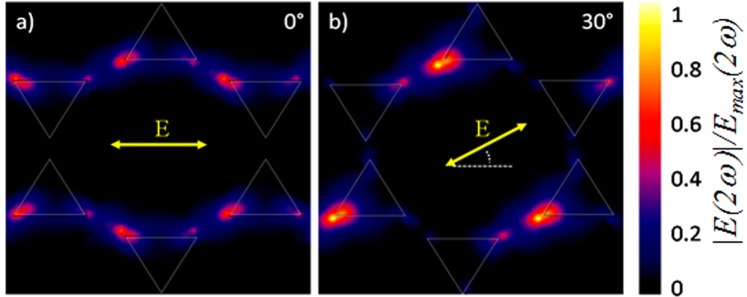
SHG electric field distribution calculated for the nanoprism array at an incidence angle of 5° for two fundamental light polarization angles: (**a**) 0° and (**b**) 30°. The field is shown normalized to its maximum value *E*_*max*_*(2ω*).

To analyse the nonlinear response at an angle of incidence of 5°, the SHG electric field distribution is plotted for two different polarization angles, 0° and 30° (Fig. 7). As it can be seen from the figure, the SHG signal is stronger at a polarization angle of 30°, thus confirming the fact that the high confinement of the fundamental field generated at every 60 degrees results in an enhancement of the SHG signal.

**Figure 8:**
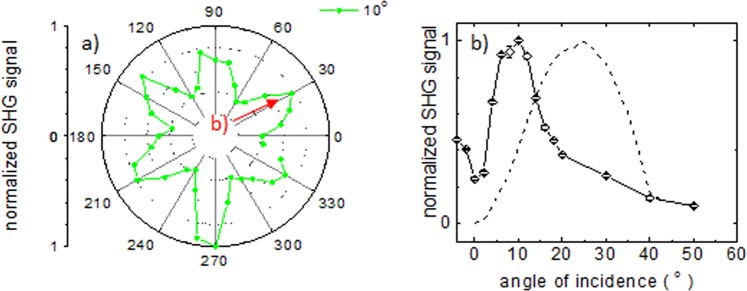
(**a**) Experimental polarization dependence of the SHG signal for an incidence angle *β* = 10 °, showing the well-defined 6-fold symmetry; and (**b**) variation of the measured SHG signal (full line) with incidence angle *β*, for a fixed polarization angle *θ* at one maximum, point (**b**) in (**a**). Also shown in (**b**) is the result of simulations for the experimental conditions employed (dashed line).

Given that we observe a 6-fold modulation in the polarization dependence of the SHG signal with a focused beam even at normal incidence, we decided to explore its dependence with incidence angle *β*. Figure 8(a) shows the SHG as a function of polarization, this time for an incidence angle of 10°, and the same input average power of 88 mW employed for the results in Fig. 3(b). The data shows again the same 6-fold modulation, with a deeper modulation contrast, which is in good agreement with the simulation results shown in Fig. 5. We then kept the polarization angle θ fixed at a value for which a maximum is observed, marked as b) in Fig. 8(a), and varied the incidence angle β, for the same input average power. Figure 8(b) shows that the signal increases with β, reaches a maximum around 10°, and then starts decreasing. A simulation conducted for the same conditions is also shown in Fig. 8(b), and qualitatively coincides with the behaviour observed, showing a well-defined maximum, albeit happening at a larger β value of 24°. This discrepancy again probably has to do with the fact that the simulations are made considering a plane wave input beam, while a focused beam is employed in the experiments. Nevertheless, these results at oblique incidence are consistent with the fact that we observed a modulation of the signal with the polarization angle when we use a focused beam even at normal incidence.

**Figure 9:**
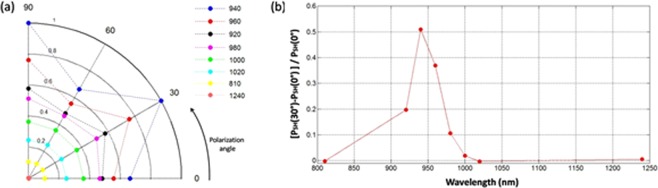
Calculated spectral dependence of the SHG contrast as a function of the polarization for oblique incidence (5° angle) around the dipolar resonance, (**a**) shows the simulated SHG values for a few polarization angle, 0°, 30°, 60°, and 90° at several wavelengths around the resonance. The wavelength dependence of the modulation depth *(P*_*SH*_*(30°)* − *P*_*SH*_*(0°))/P*_*SH*_*(0°)* is shown in (**b**).

It should be noted that in addition to the LSP-related resonant enhancement, nonresonant excitation also leads to some field enhancement at the sharp tips of the nanoprisms. Both effects lead to the local field distribution that ultimately determines the SHG efficiency. Because of this, we conducted simulations of the SHG process for different wavelengths across the dipolar and quadrupolar resonances for oblique incidence at 5°. Figure 9(a) shows the SH signals calculated for polarizations at 0°, 30°, 60°, and 90° at wavelengths around the dipolar LSPR, which display in most cases the 6-fold symmetry expected, and a lack of modulation for wavelengths away from resonance, even if there were non-vanishing signals. Figure 9(b) plots the SH power maxima to minima modulation depth, *(P*_*SH*_*(30°)* − *P*_*SH*_*(0°))/P*_*SH*_*(0°)* calculated from the simulation results, demonstrating the importance of the LSPR for the polarization modulation of the SHG signal. It is interesting to notice that the maximum modulation depth seems to happen at wavelengths slightly shorter than the actual LSPR peak, situated at 980 nm in the simulation. Exploration for wavelengths around the quadrupolar resonance at 620 nm showed a stronger signal with no appreciable variation with polarization, even under the same oblique incidence conditions.

## Conclusions

In conclusion, we have studied the SHG in a honeycomb gold nanoprism array, and its dependency on the input polarization. For such a thin metasurface, a reasonably strong signal having a well-defined 6-fold symmetry was observed, that is closely related to the microscopic structure of the material. Electromagnetic simulations showed an excellent agreement with the experimental observations, revealing the corresponding enhancement of the fundamental and the related SHG fields for polarization angles that are aligned with the 6-fold symmetry of the nanoprism array structure. Furthermore, they established that the non-normal components presented in the angular distribution of the incident light are of the key importance for the observation of the effect.
